# Associations with experience of non-fatal opioid overdose in British Columbia, Canada: a repeated cross sectional survey study

**DOI:** 10.1186/s12954-023-00912-9

**Published:** 2023-12-13

**Authors:** Max Ferguson, Paul Choisil, Jessica Lamb, Charlene Burmeister, Cheri Newman, Kurt Lock, Samuel Tobias, Lisa Liu, Jane A. Buxton

**Affiliations:** 1 British Columbia Centre for Disease Control, Vancouver, BC Canada; 2 British Centre on Substance Use, Vancouver, BC Canada; 3 School of Population and Public Health, University of British Columbia, Vancouver, BC Canada

**Keywords:** Opioid overdose, Unregulated drug poisoning emergency, Harm reduction, Fentanyl

## Abstract

**Introduction:**

Lives lost in North America due to the unregulated drug poisoning emergency are preventable and those who survive an opioid overdose may suffer long-term disability. Rates of opioid overdose more than doubled following the onset of the COVID-19 pandemic in British Columbia, Canada.

**Materials and methods:**

Our analytical sample was comprised of 1447 participants from the 2018, 2019, and 2021 Harm Reduction Client Survey who responded yes or no to having experienced an opioid overdose in the past 6 months. Participants were recruited from harm reduction sites from across British Columbia. We used logistic regression to explore associations of experiencing an opioid overdose.

**Results:**

Overall, 21.8% of participants reported experiencing an opioid overdose in the last six months (18.2% in 2019 and 26.6% in 2021). The following factors were positively associated with increased adjusted odds of experiencing a non-fatal opioid overdose: cis men relative to cis women (AOR 1.49, 95% CI 1.10–2.02), unstably housed compared to people with stable housing (AOR 1.87, 95% CI 1.40–2.50), and participants from 2021 compared to those from 2019 (AOR 3.06, 95% CI 1.57–5.97). The effects of both previous experience of a stimulant overdose and having witnessed an opioid overdose depended on the year of study, with both effects decreasing over subsequent years.

**Conclusions:**

Overdoses have increased over time; in 2021 more than one in four participants experienced an overdose. There is an urgent need for policy and program development to meaningfully address the unregulated drug poisoning emergency through acceptable life-saving interventions and services to prevent overdoses and support overdose survivors.

**Supplementary Information:**

The online version contains supplementary material available at 10.1186/s12954-023-00912-9.

## Introduction

Opioid-related overdose is causing an unprecedented number of deaths worldwide and is especially severe in North America [1]. This loss of life has been referred to as the unregulated drug poisoning emergency.^1^ Over 13,000 people have died in British Columbia due to illicit drug toxicity since a public health emergency was declared in 2016 [2]. Fentanyl in the illicit drug supply is driving the rate of deaths with fentanyl detected in more than 80% of illicit drug deaths in British Columbia since 2017 [3] and levels of fentanyl in the illicit drug supply are highly variable [4].

The COVID-19 pandemic exacerbated already dire numbers of fatal overdose [5]. In British Columbia between March and December 2020, overdose deaths more than doubled compared to the same timeframe in 2019 [5]. Following the onset of the COVID-19 pandemic, people who use drugs from across Canada reported the quality of substances decreased, the cost of substances increased and perceived that their risk of overdose had increased [6]. In a study conducted in British Columbia, more than a third of participants (36.9%) reported a decline in the quality of drugs that they most frequently used during the COVID-19 pandemic which was associated with an increased risk of overdose [7].

Many lives are lost to opioid overdose and those that survive may be disabled by the experience. Non-fatal opioid overdose is associated with severe injury and disability including brain injury (encephalopathy) due to insufficient blood flow or oxygenation to the brain which can manifest as pain, disorders of consciousness, movement disorders, epilepsy, and changes in cognitive function such as learning and memory, and changes in behaviour and emotional regulation [8, 9].

A British Columbia cross-sectional analysis of a random sample of BC residents found people who experienced a drug toxicity event, compared to those who did not, were 15 times more likely to have encephalopathy [10]. In a retrospective cohort study of administrative data in British Columbia, 3% of unintentional opioid overdose admissions included encephalopathy [11] marked by a notably altered mental state [12].

This study aims to investigate factors associated with reported experience of opioid overdose in the prior six months using survey data from British Columbia, Canada from 2018, 2019, and 2021.

## Materials and methods

Data come from the Harm Reduction Client Survey (HRCS) administered by the BC Centre for Disease Control which collects information on substance use patterns, experiences of harms and access to and use of harm reduction services. We merged data from the 2018, 2019, and 2021 HRCS for this analysis. As data does not follow the same individuals over time, this study is cross-sectional. A total of 1644 people participated in the HRCS over the 3 years. Our analytic sample is comprised of the 1447 individuals who provided information on our outcome variable: experience of an opioid overdose.

Details on the initial HRCS development [13], and methods for the 2018 [14], and 2019 [15, 16] HRCS have been documented elsewhere. The surveys can be found at http://www.bccdc.ca/health-professionals/data-reports/harm-reduction-client-survey.

For all years, a two-stage convenience sampling approach was used. Harm reduction program coordinators from the five health regions in British Columbia were consulted to identify potential participating sites, which were recruited based on willingness to participate and capacity for recruitment and data collection. Trained site staff and volunteers assisted in participant recruitment and administration of the paper-based survey. Data for the 2018 HRCS were collected May to August 2018 at 27 harm reduction sites across British Columbia; 2019 data were collected October to December 2019 at 22 sites, and data for the 2021 HRCS were collected March 2021 to January 2022 at 17 sites.

Eligibility criteria for participation in each HRCS included being 19 years or older, self-reported substance use of any illegal substance other than or in addition to cannabis in the past six months, and ability to provide verbal informed consent. Participants received $10 honorarium in 2018 and 2019 and $15 in 2021. Sites received $5/participant in compensation. Data entry and analysis occurred at the BCCDC.

### Study variables

Our outcome variable of interest was whether participants reported experiencing an opioid overdose in last 6 months (yes, no) see Additional file 1.

Sociodemographic explanatory variables included geographic health region (Fraser, Interior, Island, Northern, Vancouver Coastal), age (29 and under, 30–39, 40–49, 50 and over, unknown), gender (cis man, cis woman, trans and gender expansive, unknown), stable housing yes (in a private residence, other residence (hotels, motels, rooming houses, single room occupancy, social/supportive housing), no (have no regular place to stay (homeless, couch surf, no fixed address); in a shelter), unknown), employment (employed (full or part time work or paid volunteering), unemployed, unknown).

We included year as an explanatory variable (2018, 2019, 2021).

Drug use characteristic variables included substances reported used in the last three days; all variables were dichotomous (yes, no), including individual opioids, stimulants, benzodiazepines, cannabis or hash, tobacco, alcohol; any opioid, any stimulant, any benzodiazepine, or any polysubstance use (yes was operationalized as any combination of opioids, stimulants or benzodiazepines). Mode of consumption of any drugs or of any opioids over the last three days was included as a dichotomous explanatory variable (yes, no): smoke, snort, inject, swallow, another mode of consumption (see Table 1 for more details). Other explanatory variables include naloxone possession (yes; no, I do not have a kit but I want one; no, I do not have a kit and I do not want one; unknown), experience of stimulant overdose in the last six months (yes, no, don’t know, unknown), and witnessing an overdose in someone using any opioids in the last six months (yes, no, don’t know, unknown).

**Table 1:** Study variables stratified by experience of opioid overdose, frequencies of study variables, and bivariable regression findings

	Experience opioid overdose in last 6 months		Total (*n* = 1447) *n* (column %)	Bivariable regression *p* value
	Yes (*n* = 316) *n* (column %)	No (*n* = 1131) *n* (column %)		
Experience Opioid Overdose in Last 6 Months				
Yes			316 (21.8)	–
No			1131 (78.2)	–
*Sociodemographic Variables*				
Health Region				
Fraser	92 (29.1)	325 (28.7)	417 (28.8)	0.30
Interior	64 (20.3)	225 (19.9)	289 (20.0)	0.33
Island	58 (18.4)	201 (17.8)	259 (17.9)	0.30
Northern	57 (18.0)	184 (16.3)	241 (16.7)	0.18
Vancouver Coastal	45 (14.2)	196 (17.3)	241 (16.7)	Ref
Age				
29 and Under	67 (21.2)	184 (16.3)	251 (17.3)	** < 0.01**
30–39	94 (29.7)	298 (26.3)	392 (27.1)	0.30
40–49	78 (24.7)	297 (26.3)	375 (25.9)	**0.03**
50 and Over	72 (22.8)	331 (29.3)	403 (27.9)	Ref
Unknown	5 (1.6)	21 (1.9)	26 (1.8)	0.86
Gender				
Cis woman	91 (28.8)	412 (36.4)	503 (34.8)	Ref
Cis man	221 (69.9)	681 (60.2)	902 (62.3)	**0.01**
Transgender and gender expansive	2 (0.6)	25 (2.2)	27 (1.9)	0.17
Unknown	2 (0.6)	13 (1.1)	15 (1.0)	0.64
Stable Housing				
Yes	158 (50.0)	751 (66.4)	909 (62.8)	Ref
No	153 (48.4)	361 (31.9)	514 (35.5)	** < 0.01**
Unknown	5 (1.6)	19 (1.7)	24 (1.7)	0.66
Employment				
Employed	53 (16.8)	245 (21.7)	298 (20.6)	Ref
Unemployed	248 (78.5)	841 (74.4)	1089 (75.3)	0.07
Unknown	15 (4.7)	45 (4.0)	60 (4.1)	0.69
*Year*				
2018	86 (27.2)	320 (28.3)	406 (28.1)	0.25
2019	102 (32.3)	458 (40.5)	560 (38.7)	Ref
2021	128 (40.5)	353 (31.2)	481 (33.2)	** < 0.01**
*Last 3 Day Drug Use Reported*				
*Opioids*				
Methadone				
Yes	91 (28.8)	292 (25.8)	383 (26.5)	Ref
No	225 (71.2)	839 (74.2)	1064 (73.5)	0.289
Morphine				
Yes	68 (21.5)	129 (11.4)	197 (13.6)	** < 0.01**
No	248 (78.5)	1002 (88.6)	1250 (86.4)	Ref
Hydromorphone (Dilaudid)				
Yes	61 (19.3)	101 (8.9)	162 (11.2)	** < 0.01**
No	255 (80.7)	1030 (91.1)	1285 (88.8)	Ref
Oxycodone				
Yes	17 (5.4)	35 (3.1)	52 (3.6)	0.06
No	299 (94.6)	1096 (96.9)	1395 (96.4)	Ref
Fentanyl				
Yes	204 (64.6)	478 (42.3)	682 (47.1)	** < 0.01**
No	112 (35.4)	653 (57.7)	765 (52.9)	Ref
Diacetylmorphine (Heroin)				
Yes	192 (60.8)	445 (39.3)	637 (44.0)	** < 0.01**
No	124 (39.2)	686 (60.7)	810 (56.0)	Ref
*Stimulants*				
Cocaine powder				
Yes	65 (20.6)	191 (16.9)	256 (17.7)	0.13
No	251 (79.4)	940 (83.1)	1191 (82.3)	Ref
Crack cocaine				
Yes	89 (28.2)	251 (22.2)	340 (23.5)	**0.03**
No	227 (71.8)	880 (77.8)	1107 (76.5)	Ref
Crystal Methamphetamine				
Yes	245 (77.5)	763 (67.5)	1008 (69.7)	** < 0.01**
No	71 (22.5)	368 (32.5)	439 (30.3)	Ref
Other Stimulants (e.g. Ritalin/Adderall)				
Yes	32 (10.1)	64 (5.7)	96 (6.6)	** < 0.01**
No	284 (89.9)	1067 (94.3)	1351 (93.4)	Ref
*Benzodiazepines*				
Alprazolam (Xanax)				
Yes	19 (6.0)	30 (2.7)	49 (3.4)	** < 0.01**
No	297 (94.0)	1101 (97.3)	1398 (96.6)	Ref
Other Benzodiazepine (e.g. Ativan/Valium)				
Yes	67 (21.2)	135 (11.9)	202 (14.0)	** < 0.01**
No	249 (78.8)	996 (88.1)	1245 (86.0)	Ref
*Cannabis, Tobacco, Alcohol*				
Cannabis or Hash				
Yes	162 (51.3)	560 (49.5)	722 (49.9)	0.58
No	154 (48.7)	571 (50.5)	725 (50.1)	Ref
Tobacco				
Yes	253 (80.1)	881 (77.9)	1134 (78.4)	0.41
No	63 (19.9)	250 (22.1)	313 (21.6)	Ref
Alcohol				
Yes	117 (37.0)	434 (38.4)	551 (38.1)	0.66
No	199 (63.0)	697 (61.6)	896 (61.9)	Ref
*Last 3 Day Use of Drugs by Category*				
Any Opioid				
Yes	266 (84.2)	684 (60.5)	950 (65.7)	** < 0.01**
No	50 (15.8)	447 (39.5)	497 (34.3)	Ref
Any Stimulant				
Yes	267 (84.5)	887 (78.4)	1154 (79.8)	** < 0.01**
No	49 (15.5)	244 (21.6)	293 (20.2)	Ref
Any Benzodiazepine				
Yes	75 (23.7)	147 (13.0)	222 (15.3)	**0.02**
No	241 (76.3)	984 (87.0)	1225 (84.7)	Ref
Any Polysubstance Use				
Yes	244 (77.2)	603 (53.3)	847 (58.5)	** < 0.01**
No	72 (22.8)	528 (46.7)	600 (41.5)	Ref
*Mode of Consumption for Any Drugs over Last 3 Days*				
Smoke Any Drug				
Yes	221 (69.9)	691 (61.1)	912 (63.0)	** < 0.01**
No	95 (30.1)	440 (38.9)	535 (37.0)	Ref
Snort Any Drug				
Yes	123 (38.9)	420 (37.1)	543 (37.5)	0.56
No	193 (61.1)	711 (62.9)	904 (62.5)	Ref
Inject Any Drug				
Yes	173 (54.7)	466 (41.2)	639 (44.2)	** < 0.01**
No	143 (45.3)	665 (58.8)	808 (55.8)	Ref
Swallow Any Drug				
Yes	147 (46.5)	360 (31.8)	507 (35.0)	** < 0.01**
No	169 (53.5)	771 (68.2)	940 (65.0)	Ref
Consume Any Drug by Another Mode of Consumption				
Yes	36 (11.4)	141 (12.5)	177 (12.2)	0.61
No	280 (88.6)	990 (87.5)	1270 (87.8)	Ref
*Mode of Consumption for Opioids over Last 3 Days*				
Smoke Any Opioid				
Yes	174 (55.1)	421 (37.2)	595 (41.1)	** < 0.01**
No	142 (44.9)	710 (62.8)	852 (58.9)	Ref
Snort Any Opioid				
Yes	78 (24.7)	224 (19.8)	302 (20.9)	0.06
No	238 (75.3)	907 (80.2)	1145 (79.1)	Ref
Inject Any Opioid				
Yes	138 (43.7)	300 (26.5)	438 (30.3)	** < 0.01**
No	178 (56.3)	831 (73.5)	1009 (69.7)	Ref
Swallow Any Opioid				
Yes	128 (40.5)	298 (26.3)	426 (29.4)	** < 0.01**
No	188 (59.5)	833 (73.7)	1021 (70.6)	Ref
Consume Any Opioid by Another Mode of Consumption				
Yes	24 (7.6)	102 (9.0)	126 (8.7)	0.43
No	292 (92.4)	1029 (91.0)	1321 (91.3)	Ref
*Naloxone Possession*				
Yes	227 (71.8)	800 (70.7)	1027 (71.0)	0.66
No, I do not have a kit but I want one	48 (15.2)	169 (14.9)	217 (15.0)	0.72
No, I do not have a kit and I do not want one	31 (9.8)	120 (10.6)	151 (10.4)	Ref
Unknown	10 (3.2)	42 (3.7)	52 (3.6)	0.84
*Overdose*				
Experience Stimulant Overdose in Last 6 Months				
Yes	98 (31.0)	72 (6.4)	170 (11.7)	** < 0.01**
No	199 (63.0)	1010 (89.3)	1209 (83.6)	Ref
Don’t know	12 (3.8)	19 (1.7)	31 (2.1)	** < 0.01**
Unknown	7 (2.2)	30 (2.7)	37 (2.6)	0.69
Witness an Opioid Overdose in Last 6 Months				
Yes	232 (73.4)	630 (55.7)	862 (59.6)	** < 0.01**
No	64 (20.3)	425 (37.6)	489 (33.8)	Ref
Don’t know	4 (1.3)	18 (1.6)	22 (1.5)	0.49
Unknown	16 (5.1)	58 (5.1)	74 (5.1)	**0.05**

Data sourced from the British Columbia Harm Reduction Client Survey (2018, 2018, and 2021)

### Analysis

We completed frequency tables for all study variables and cross tabulation for all explanatory variables stratified by our outcome variable, experience of opioid overdose in the past six months. We used a hierarchical approach with demographic, year, and drug use characteristic blocks. Likelihood ratio tests were utilized to see if each block significantly improved the amount of outcome variability explained by the model.

We used a purposeful model building approach: Individual and categories of substances reported used and mode of consumption in last three days were included in bivariable regression but not included in the model building (outcome variable was in past six months so likely preceded past three day drug use); all other explanatory variables with at least one level with a p-value of 0.25 or less in bivariable regression were assessed as candidates for inclusion in the final model [17]. We subsequently used backwards selection for each block (demographics, year, and drug use characteristics) based on significantly decreasing Akaike Information Criterion values [17]. We then evaluated the presence of effect modification by year of survey on respective variables in the final model and confirmed significance and improved model fit with likelihood ratio tests.

We worked with Professionals for the Ethical Engagement of Peers (PEEP), a consultation and advisory board that provides guidance on harm reduction policy and research from the perspective of those with lived and living experience of drug use. PEEP advised on the analytical plan prior to analysis and provided feedback on the study results and interpretations using their real-world observations to ensure reporting of results was not stigmatizing.

## Results

Our analytic sample was comprised of the 1447 participants who responded yes or no to having experienced an opioid overdose in the past six months.

### Findings

Table 1 provides an overview of the 1447 study participants, of these 316 (21.8%) reported experiencing an opioid overdose and 170 (11.7%) reported a stimulant overdose in the past six months. More than half of participants (*n* = 862, 59.6%) reported having witnessed an overdose in the same period.

A majority of participants were cis men (*n* = 902, 62.3%), had stable housing (*n* = 909, 62.8%) and were unemployed (*n* = 1089, 75.3%). The most common opioids reported used in the last three days were fentanyl (47.1%, *n* = 682) and heroin (44%, *n* = 637). Crystal methamphetamine was the most frequently used stimulant in the last three days reported by 69.7% of participants (*n* = 1008). Polysubstance use was frequently reported; 58.5% of participants (*n* = 847) used at least two of the three drug categories (opioids, stimulants, benzodiazepines) in the last 3 days.

### Model building

In the sociodemographic block (block 1) we tested the following variables for inclusion in the final model: health region, age, gender, stable housing, and employment. Gender and stable housing improved model fit and were included. We included age due to conceptual relevance despite the variable not significantly improving model fit. Year, the only variable within block 2, was evaluated and found to improve model fit. See Additional file 1: Table S1 for block 1 and block 2 results.

Within the drug use characteristics block (block 3) overdose experience in the last six months (experience of stimulant overdose and witnessing an overdose) were evaluated for inclusion in the final model. Likelihood ratio tests showed that the block 2 model with year was a significant improvement on the goodness of fit compared to the block 1 model with sociodemographic variables only (*p* value: < 0.01) and the block 3 model with drug use characteristics was a significant improvement compared to the block 2 model with year and sociodemographic variables (*p* value: < 0.01).

Effect modification by year was examined by evaluating all covariates and their respective interaction with year of survey within the block 3 model. Both experience of stimulant overdose and witnessing an overdose were found to be significant and the inclusion of both interaction terms was found to improve model fit with likelihood ratio tests (*p* value < 0.01).

Table 2 shows the unadjusted odds ratios and final model in which gender, housing stability, year, experiencing a stimulant overdose in the last six months, and witnessing an overdose in the last 6 months were all significantly associated with experiencing an opioid overdose in the last 6 months. Interaction terms of experiencing a stimulant overdose and year, and witnessing an overdose and year showed that these effects depend on the year of survey. The strength of effect for these variables decreased in subsequent years of the survey.

**Table 2:** Unadjusted and final hierarchical logistic regression model results for the association with experience of opioid overdose

			Block 3 (final model): drug use characteristic with effect modification by year of survey	
	Unadjusted odds ratio (95% confidence interval)	*p* value	Adjusted odds ratio (95% confidence interval)	*p* value
*Age category*				
29 and Under	**1.67 (1.15–2.44)**	**0.01**	1.53 (0.99–2.36)	0.05
30–39	**1.45 (1.03–2.05)**	**0.03**	1.19 (0.81–1.77)	0.37
40–49	1.21 (0.85–1.72)	0.30	1.01 (0.68–1.51)	0.95
50 and Over	Ref	Ref	Ref	Ref
Unknown	1.09 (0.40–3.00)	0.86	1.17 (0.38–3.63)	0.79
*Gender*				
Cis Woman	Ref	Ref	Ref	Ref
Cis Man	**1.47 (1.12–1.93)**	**0.01**	**1.49 (1.10–2.02)**	**0.01**
Transgender and gender expansive	0.36 (0.08–1.55)	0.17	0.45 (0.09–2.23)	0.34
Unknown	0.70 (0.15–3.14)	0.64	0.82 (0.16–4.16)	0.81
*Stable housing*				
Yes	Ref	Ref	Ref	Ref
No	**2.01 (1.56–2.60)**	** < 0.01**	**1.87 (1.40–2.50)**	** < 0.01**
Unknown	1.25 (0.46–3.40)	0.66	1.18 (0.35–3.95)	0.79
*Year*				
2018	1.21 (0.88–1.66)	0.25	0.69 (0.29–1.64)	0.40
2019	Ref	Ref	Ref	Ref
2021	**1.63 (1.21–2.19)**	** < 0.01**	**3.06 (1.57–5.97)**	** < 0.01**
*LAST 6 Months*				
*Experience Stimulant Overdose*^a^				
Yes	**6.91 (4.92–9.71)**	** < 0.01**		
No	Ref	Ref		
Don’t Know	**3.21 (1.53–6.71)**	** < 0.01**		
Unknown	1.18 (0.51–2.73)	0.69		
*2018*				
Yes	–		**18.85 (9.17–38.75)**	** < 0.01**
No	–		Ref	Ref
Don’t Know	–		**38.40 (3.36–438.75)**	** < 0.01**
Unknown	–		2.22 (0.51–9.70)	0.29
*2019*				
Yes	–		**8.58 (4.81–15.33)**	** < 0.01**
No	–		Ref	Ref
Don’t Know	–		1.00 (0.21–4.81)	1.0
Unknown	–		2.94 (0.47–18.33)	0.25
*2021*				
Yes	–		**2.73 (1.42–5.24)**	** < 0.01**
No	–		Ref	Ref
Don’t Know	–		**9.23 (1.81–47.24)**	** < 0.01**
Unknown	–		0.49 (0.10–2.39)	0.38
*Witness Overdose*^a^				
Yes	**2.45 (1.81–3.31)**	** < 0.01**		
No	Ref	Ref		
Don’t Know	1.48 (0.48–4.50)	0.49		
Unknown	1.83 (0.99–3.38)	0.05		
*2018*				
Yes	–		**3.35 (1.58–7.10)**	** < 0.01**
No	–		Ref	Ref
Don’t Know	–		0.14 (0.01–2.35)	0.17
Unknown	–		**4.93 (1.37–17.67)**	**0.01**
*2019*				
Yes	–		**2.63 (1.50–4.62)**	** < 0.01**
No	–		Ref	Ref
Don’t Know	–		0.00 (0.00-Inf)	0.98
Unknown	–		1.62 (0.56–4.66)	0.37
*2021*				
Yes	–		1.49 (0.91–2.44)	0.12
No	–		Ref	Ref
Don’t Know	–		2.35 (0.43–12.80)	0.32
Unknown	–		0.47 (0.09–2.43)	0.37

Bolded results indicate significance at *p* < 0.05

^a^The Block 3 Model (Final Model) includes interaction terms for experience of stimulant overdose * year and witnessed an overdose * year

Data sourced from the British Columbia Harm Reduction Client Survey (2018, 2019, and 2021)

Cis men had 1.49 times the adjusted odds of experiencing opioid overdose in the last six months relative to cis women (95% CI 1.10–2.02). Participants without stable housing had 1.87 times the adjusted odds of experiencing opioid overdose relative to their stably housed peers (95% CI 1.40–2.50). Those who participated in the survey in 2021 had 3.06 times the adjusted odds of experiencing overdose in the last six months relative to those who participated in 2019 (95% CI 1.57–5.97). In the year 2018, those who had experienced a stimulant overdose had 18.85 times the odds of experiencing an opioid overdose (95% CI 9.17–38.75), compared to those who did not within the same year. This effect decreased in 2019 and further decreased in 2021 (AOR 2021: 2.73, 95% CI 1.42–5.24). Similarly, those who witnessed an overdose in 2018 had 3.35 times the odds of experiencing an overdose themselves (95% CI 1.58–7.10) compared to those who did not within the same year. This effect decreased over subsequent years. There was no significant effect of witnessing an overdose on experiencing one personally within 2021 (AOR 2021: 1.49, 95% CI 0.91–2.44).

We assessed collinearity by looking at variance inflation factor (VIF) and determined that most variables fell within acceptable limits as their VIF were all below 4 [18]. The variable for witnessing an overdose had a notably higher VIF (12.4), indicating potential collinearity for this specific variable.

## Discussion

We found that experience of opioid overdose in the last six months was significantly associated with identifying as cis man, unstable housing, participating in the 2021 HRCS, experiencing a stimulant overdose in the previous six months, and witnessing an overdose in the previous six months in the 2018 and 2019 but not 2021 surveys. Our findings on associations with experience of opioid overdose offer opportunities to guide public health practice to prevent loss of life.

Our finding that those without stable housing had increased odds of experiencing overdose corresponds with Public Health Agency of Canada report that people experiencing homelessness are overrepresented among those who died of acute drug or alcohol toxicity in Canada in 2016–2017 [19]. In contrast, in British Columbia the majority of deaths occur in private or other residences [2]. Papamihali et al. [14] found reasons for using substances alone in British Columbia were for convenience and comfort, however, those who do not have stable housing may use in places where they are more likely to be observed if they experience an overdose, and can be resuscitated by by-standers and emergency services called, hence may be more likely to survive an overdose.

We found a lower proportion of participants reported overdoses in 2019 (18.2%) relative to 2018 (21.2%) and 2021 (26.6%); which aligns with the rate of overdose deaths reported in British Columbia: 19.3 per 100,000 population in 2019, 31.2/100,000 in 2018, and 43.6/100,000 in 2021 [2]. In prior iterations of the HRCS rates of reported overdose was considerably lower; 8% of participants reported experiencing an opioid overdose in 2014 [20]; and 13% in 2015 [21]. Our findings align with previous research stating that rates of fatal overdose increased during the COVID-19 pandemic [2, 5].

People who have experienced an overdose continue to use opioids; we found 84.2% of people who experienced opioid overdose in the last six months report using opioids in the last three days. Therefore to prevent subsequent overdose it is important to separate people from the toxic unregulated market by providing a regulated supply of opioids such as opioid agonist therapy (OAT) and safer supply.

OAT provides non-psychoactive substances such as methadone and buprenorphine/naloxone to people who use opioids to help people manage opioid withdrawal symptoms [22]. In British Columbia, 12-month retention rates for OAT range between 7.9 and 18.1% depending on medication and only two-thirds of people accessing OAT services reached the minimum effective dose at any point in their treatment episode [22]. Additionally, many people continue to access the illicit supply while on OAT and therefore continue to risk overdose [23]. While some benefit from OAT, it is not an appropriate or acceptable treatment for all.

Safer supply programs provide a legal and regulated supply of drugs with psychoactive properties to those who use them [24]. Safer supply interventions do not aim to change people who use drugs into those who do not use drugs, but provides an alternative to the unpredictable and dangerous illicit supply.

The association between witnessing and experiencing opioid overdose has been noted elsewhere [25, 26]. People in the same community may be exposed to the same toxic drug supply or use the same toxic batch of drugs together, and thus experience similar overdose outcomes. Harm reduction messaging advises not to use alone and if using drugs with others to stagger use so that someone is able to respond to an overdose should it occur. Our finding that the effect of witnessing an overdose diminished over subsequent years of the survey may be due to the increased prevalence of having witnessed an overdose, but should be further explored.

Association with past six month experience of stimulant overdose had the greatest effect measure in our study; a third of people (31.0%, *n* = 98) who had experienced an opioid overdose in the past six months also experienced a stimulant overdose. In response to input received from people who use substances, to help differentiate a stimulant overdose from an opioid overdose due to opioid contamination of the stimulant, we added “(overamped)” to the survey question. Intentional polysubstance use is common and known to be associated with opioid overdose [27]. People who use drugs in BC report various reasons for concurrent use of stimulants and opioids which include self-medication (e.g., to manage mental health problems or pain), availability and preference, drug effects/properties, and financial and life situation (e.g., better value and more able to complete desirable activities), and a small number of participants reported concurrent use to avoid overdose [16].

Individuals who report recent-non fatal overdose have an elevated risk of death from a subsequent overdose [28]. While participants in this study lived to report their experience of overdose, it is crucial to implement timely and meaningful interventions in response to increasing overdoses to prevent a subsequent overdose.

Take-Home Naloxone programs are life-saving interventions that enable people to reverse opioid overdose in the community [29]. While naloxone is an important tool for saving lives of friends, family, and community members it is not a sufficient response to stem the loss of life [30]. Naloxone can save the life of someone already experiencing overdose, but does not prevent overdose or the trauma and potential injury associated with it.

People with lived and living experience of substance use are the forefront of the unregulated drug poisoning emergency [30, 31], and carry the burden of saving lives of friends, family, and other community members [32]. Responders often experience overwhelming loss, burnout, and frustration, and severe grief and loss responses are associated with experiencing or witnessing an overdose [32, 33]. People who are exposed to overdose may exhibit symptoms of Post-Traumatic Stress Disorder and lack support and debriefing services [34, 35].

### Limitations and strengths

Self-reported experience of opioid overdose is a poor measure of incidence of opioid overdose due to survival bias; tragically many people experiencing opioid overdose were not captured in this sample as they died. We used self-reported drug use which was not confirmed by urinalysis, participants may be unaware of content of their drugs which may change over time. For example there was an increased adulteration of fentanyl with benzodiazepines in 2021 compared to previous years [36]. Literature shows that overdose deaths are associated with benzodiazepine co-involvement [37, 38]. Benzodiazepines depress breathing and can work synergistically with opioids [38]. Co-involvement of benzodiazepine may also complicate overdose resuscitation as those overdosing may not regain consciousness with naloxone causing some responders to administer more naloxone and precipitate opioid withdrawal [39].

Additionally, the study may be vulnerable to self-report bias, especially considering the traumatizing nature of overdose. Another limitation of the HRCS is that data on other known factors related to overdose such as recent release from prison were not collected. Participants were recruited from harm reduction sites so were likely to be using illegal substances despite experiencing a previous overdose or being prescribed OAT. As the study was cross-sectional we were unable to confirm temporality or causation, also substance use was collected for the last 3 days while the overdose occurred sometime in the past 6 months.

No identifying information was recorded in the HRCS so it is possible that the same individual participated in repeat iterations of the survey. Since our outcome variable of experience of opioid overdose is specific to the six months prior to the survey and the surveys took place at least a year apart, this should not affect the validity of our primary variable of interest.

A strength of our study is that survey data allows participants to provide information based on their current situation at the time of the surveys before and after the advent of COVID-19, for example, the HRCS collects details on gender, housing, employment, experiences of overdose and substance use and these data may not be recorded in administrative or coroner’s data. Therefore findings can identify the extent of emerging issues to inform timely public health interventions. In addition, the engagement of PEEP throughout the study ensures the analysis and interpretation of the findings is relevant to the context and reality of people who use substances.

## Conclusions

A great many lives have been lost during the unregulated drug poisoning emergency and those who survive an opioid overdose may suffer long-term disability. In our study more than one in four participants in 2021 experienced an overdose in the last six months with overdose experience associated with being cis man, unstably housed, recent year, experiencing a stimulant overdose and witnessing an overdose in the last 6 months. This high rate of overdose is a call to action to implement life-saving interventions that are accessible and acceptable to those at risk of an opioid overdose including increased access to observed consumption sites, opioid agonist treatment and safer supply. Interventions aimed at supporting overdose survivors should be improved such as peer based mental health supports and assessment, and support for those disabled by their overdose.

### Supplementary Information

**Additional file 1**. Development of opioid overdose question.

## Data Availability

The datasets used and/or analysed during the current study are available from the corresponding author upon reasonable request.

## Footnotes

1 We use the term “unregulated drug poisoning emergency” based on the recommendation of Professionals for the Ethical Engagement of Peers and other stakeholders in BC. This language links deaths to the absence of a regulated supply of drugs and highlights that harms of illegal opioids and adulterants in the toxic unregulated supply and the declaration of the public health emergency in BC in 2016. This term replaces “opioid toxicity crisis” or “overdose crisis”.
