# Supplementary material for: Associations with experience of non-fatal opioid overdose in British Columbia, Canada: a repeated cross sectional survey study
Source: Harm Reduct J. 2023 Dec 13;20:178. doi: 10.1186/s12954-023-00912-9 (PMC10717189; doi:10.1186/s12954-023-00912-9)
Supplement: Supplementary file 1 — Additional file 1. Development of opioid overdose question. [file 12954_2023_912_MOESM1_ESM.docx]

**Supplemental 1.**

**Development of opioid overdose question in the BC Harm Reduction Client Survey**

All survey questions received input from people with lived and living experience (PWLLE) in development. The questions were piloted with PWLLE and input incorporated before the survey was rolled out. The question regarding opioid overdose was thought to be clear and not need additional definition. This was in the light of the frequency of opioid overdoses occurring across British Columbia due to the prevalence of fentanyl and analogues (90%) in the unregulated opioid supply.

In all three years more than 50% of respondents reported witnessing an opioid overdose.^1^ In addition the majority of survey participants carry naloxone kits (74% in 2021) and thus would have received overdose recognition and response training.^1^

**Wording of opioid overdose question in 2018, 2019 and 2021 surveys**

The question varied slightly between years as awareness of fentanyl in the unregulated drug supply increased;^1^ in 2018 39% of participants reported known fentanyl use and 22% had unknown fentanyl use.^2^

**2018:** *“In the last 6 months have YOU overdosed by accident from using any opioids, such as heroin or morphine?*

**2019:** *“In the last 6 months have YOU overdosed by accident from using any opioids (e.g. fentanyl, heroin)?*”

**2021:** *“In the last 6 months have YOU overdosed by accident from using opioids (e.g. fentanyl, heroin)?*”

**Response options for all three surveys were identical:**

(Select one) ❑ Yes ❑ No ❑ Don’t know ❑ Prefer not to say

**References**

1. Reported Substance Use, Overdose and Naloxone Kit Ownership 2012-2021. Vancouver, BC. BC Centre for Disease Control. Available at <http://www.bccdc.ca/Health-Professionals-Site/Documents/Harm-Reduction-Reports/HRCS%20Narrative%20Graphs%20%281%29.pdf>
2. Karamouzian M**,** Papamihali K, Graham B, Crabtree A, Mill C, Kuo M, Young S, Buxton JA​. Known fentanyl use is increasing among people who use drugs in British Columbia, Canada: Findings of the BC Harm Reduction Clients’ Survey. Int J Drug Policy (2020) (77) 102665 <https://www.ncbi.nlm.nih.gov/pubmed/31962283>

Supplemental Table 1: Unadjusted and initial hierarchical logistic regression model results for the association with experience of opioid overdose. Data sourced from the British Columbia Harm Reduction Client Survey (2018, 2019, and 2021).

|  |  |  | Block 1:  Socio-demographic | | Block 2:  Year | |
| --- | --- | --- | --- | --- | --- | --- |
|  | Unadjusted Odds Ratio (95% Confidence Interval) | P-Value | Adjusted Odds Ratio (95% Confidence Interval) | P-Value | Adjusted Odds Ratio (95% Confidence Interval) | P-Value |
| **Age Category** |  |  |  |  |  |  |
| 29 and under | **1.67 (1.15-2.44)** | **0.01** | **1.52 (1.03-2.25)** | **0.03** | **1.63 (1.1-2.42)** | **0.01** |
| 30-39 | **1.45 (1.03-2.05)** | **0.03** | 1.32 (0.93-1.88) | 0.12 | 1.38 (0.97-1.98) | 0.07 |
| 40-49 | 1.21 (0.85-1.72) | 0.30 | 1.18 (0.82-1.7) | 0.36 | 1.22 (0.85-1.75) | 0.29 |
| 50 and over | Ref | Ref | Ref | Ref | Ref | Ref |
| Unknown | 1.09 (0.40-3.00) | 0.86 | 1.25 (0.44-3.59) | 0.68 | 1.20 (0.42-3.46) | 0.73 |
| **Gender** |  |  |  |  |  |  |
| Cis Woman | Ref | Ref | Ref | Ref | Ref | Ref |
| Cis Man | **1.47 (1.12-1.93)** | **0.01** | **1.53 (1.16-2.02)** | **<0.01** | **1.54 (1.17-2.04)** | **<0.01** |
| Transgender and gender expansive | 0.36 (0.08-1.55) | 0.17 | 0.45 (0.11-1.97) | 0.29 | 0.45 (0.1-1.96) | 0.29 |
| Unknown | 0.70 (0.15-3.14) | 0.64 | 0.78 (0.16-3.69) | 0.75 | 0.75 (0.16-3.56) | 0.71 |
| **Stable Housing** |  |  |  |  |  |  |
| Yes | Ref | Ref | Ref | Ref | Ref | Ref |
| No | **2.01 (1.56-2.60)** | **<0.01** | **1.91 (1.47-2.48)** | **<0.01** | **1.84 (1.41-2.39)** | **<0.01** |
| Unknown | 1.25 (0.46-3.40) | 0.66 | 1.33 (0.48-3.7) | 0.58 | 1.38 (0.5-3.84) | 0.54 |
| **Year** |  |  |  |  |  |  |
| 2018 | 1.21 (0.88-1.66) | 0.25 |  |  | 1.19 (0.86-1.65) | 0.30 |
| 2019 | Ref | Ref |  |  | Ref | Ref |
| 2021 | **1.63 (1.21-2.19)** | **<0.01** |  |  | **1.63 (1.20-2.20)** | **<0.01** |
| Bolded results indicate significance at p<0.05. | | | | | | |
